# The Standard of the National Board of Dental Examiners

**Published:** 1898-02

**Authors:** 


THE
AMERICAN JOURNAL
—OF—
DENTAL SCIENCE.

VOL. XXXI. BALTIMORE, FEBRUARY, 1898. NO. 10.

ARTICLE I.

### THE STANDARD OF THE NATIONAL BOARD OF DENTAL EXAMINERS.

In this number we publish the circular letter of The Committee on Schools of The National Board of Dental Examiners, with some of the correspondence which it has elicited. We do this because we believe it to be matter that is of general professional interest, which cannot be too widely circulated. Every dentist should have placed before him all the facts, that he may be able to judge as to which side is right, and which course will best subserve the interests of higher education. Each of the three college answers presents some special view of the subject, and so all are printed. That of the Chicago College of Dental Surgery contains matter that is especially worthy careful consideration. It is a fact that some of the State Boards have licensed to practice Freshmen students, and those who have been " plucked " by the colleges. They have granted certificates to those who were too ignorant and uneducated to be received into any respectable dental college, and who had been refused matriculation. In this direction lies enough of needed work to occupy the exclusive attention of the National Association of Dental Examiners for years to come. Let that body expend some of its surplus energy in defining the duties and raising the standard of the State Boards. Then will they be more attentively listened to when the members desire to be received as the censors and supervisors of the colleges. Soldiers are not placed in command of an army until they have demonstrated their capacity in subordinate positions, and shown that they are fit to exercise authority. The present agitation, brought about by the unwarranted action of the Examiners Association, will certainly eventuate in good, for it has called attention to the great deficiencies and imperfections of that body. It must reform itself in some particulars, or it will never again be received, even as the coadjutor of the National Association of Dental Faculties, or be listened to with the respect that it should command. Perhaps, as Prof. Brophy suggests, the National Association of Faculties will mark out a course for it to pursue, but it will propably be only at its own request, for there is no disposition to follow the disrespectful and vicious precedent which it has established.

The Secretary of the Committee on Schools of the Board of Examiners seems surprised that the colleges which have a respectable standard for qualification of matriculants should decline to accept that which is presented to them. He does not appear to comprehend that the objection is not to the requirements, but to the assumption of the right of an Association like that of the Examiners to *demand* the acceptance of any standard whatever from the National Association of College Faculties. The requirements themselves are below those of the colleges whose answers are printed. Yet they cannot be accepted, because that would admit the right of the Examiners Association to impose conditions upon the colleges. This prerogative once conceded to a body in which the colleges have no representation, might be but an entering wedge for the grossest kind of abuses. There is no knowing where the exactions would end. The accountability of the colleges to any body whatever outside those acting under the responsibility of the courts of law, carries with it the gravest dangers to their integrity, because it threatens unknown and unforeseen calamities. This does not seem to have been perceived by some who have counseled acceptance. The patriots who threw overboard the tea in Boston harbor were severely condemned by certain classes of the people for taking such violent measures to evade the imposition of an insignificant tax. It would have been so much easier to pay the small sum demanded than to raise a war of rebellion. But if compliance had then been made, there would have been no American nation, and the people of England, as well as of this country, would have been the slaves of an absolute monarchy, for one aggression would but have led to another. It is in this view that the colleges have resisted the impudent attempts of the Examiners to impose a standard upon them, and to dictate as to the methods in which they shall be conducted.

The schools do not demand exemption from legal restrictions. They only protest against the domination of irresponsible bodies. There cannot be found an instance in which a college, or a college faculty, has in any way whatever attempted to resist even stringent legal restrictions, provided they were in any sense fair. College men have been foremost in moving for proper legal supervision. They desire that their privileges shall have clear legal expression. It is not true that college professors desire to graduate unqualified men, or to launch upon the profession a horde of mere pretenders to professional knowledge. Our teachers are no more dishonest than are other dentists. True, they do not all have the same conception of what should be the preliminary requirements. Some of them, unfortunately, were without a liberal preliminary education, and have raised themselves to their present position by force of talent, rather than training. But no one has the right to assume that they are otherwise than sincere and honest in their belief that the standard which many of us think essential to the best interests of dentistry is not demanded. Our teachers, as a whole, are earnestly endeavoring to raise the standard of preliminary qualifications, and the men who are without literary training are steadily being pushed to the wall, and must soon give way to better qualified men. As a whole, they are, notwithstanding the unnecessary sneers of the Secretary of the Committee of Schools, quite as honestly desirous of elevating our educational standard as are the members of the Examining Boards, and they know a great deal better how it can be done.

Another reason why the standard of the Examiners could not be accepted, was because of the arrogant and offensive way in which the movement was conducted. So long as the Examiners were willing to work as the confreres and coadjutors of the Faculties, their assistance was gladly received. But they *demanded* immediate and radical action. They could not see the difficulties which beset the Faculties, and when the movements were not as prompt and decided as they thought they should be, they accused the Faculties of insincerity, and attempted compulsion. Their own body is not composed of men of such high standing as to entitle it to dictate to everyone else. They have no standard of qualification for their own members, and fully one-half of them are not college graduates, and know nothing whatever of the college curriculum. A considerable proportion of those who have degrees, obtained them *sinecurriculo.* The Secretary of the Committee of Schools, who is most persistent in demanding the acceptance of the standard which the Examiners have endeavored to impose, is not a member of any State Board, nor eligible to membership in the National Association of Examiners. The assurance which prompts the attempt to force the colleges into acceptance as a dictator and a governor of one entirely outside all educational organizations, a man who has made himself unnecessarily obnoxious to the schools, is amazing, and is liable to the interpretation that the Examiners are prepared to go any lengths in their exactions, if once their authority is conceded.

Since the refusal of a considerable number of the colleges to accept the terms offered by the Association of Dental Examiners, there has been a denial that any domination was ever intended, or that a resort to compulsory measures was contemplated. But the following extracts from the report of the meeting at which the rules were adopted, would seem to show that it was with positive threats of punishment in case of refusal to conform that they were considered:

Rule 8. To be continued on the list of recognized colleges, all colleges must maintain these rules and conditions.

Rule 9. To more fully enforce these rules it is hereby directed that any college violating these rules shall be taken from the list of recognized colleges.

Dr. Donnelly: How would you enforce this rule?

Dr. G. Carelton Brown (Chairman Committee on Colleges): Simply by sending this resolution to each of the colleges on our list, asking them to subscribe to it, and refusing to put any college on our list who will not subscribe to it.

It is a little humiliating for teachers to be asked to accept a code of rules containing so many grammatical errors and couched in such execrable English as those submitted, especially when they emanate from men who assume pedagogical airs, but to be supposed that the members of the Association comprehended what was meant by the language employed. The above quoted extracts would seem to indicate that the Secretary of the Committee on Schools must have been quite in the dark as to their true meaning, or that he was otherwise confused, when in his answer to the Buffalo communication he says we "must repudiate that we ever said or implied that in case of non-acceptance of our regulations, yours or any other institution would be branded as unrecognized."

There is a dislike on the part of the Faculties to comply with the demands made, because it is not felt that the Examiners Association is at all national in its character. When there are representatives of but a comparatively few of the States in attendance, and when the movement is apparently dominated by those from but two or three, and they by no means the most important, when the men of national reputation and those who are accepted as truly representative are conspicuous by their absence, when such States as New York, Michigan, Illinois and others, pointedly decline to become identified with such a movement, the colleges would simply make an exhibition of their own inanity were they to accept the Board of Examiners as the supreme authority in dentistry.

When a rigid standard of preliminary qualification is adopted, it must be the result of development. The profession must grow up to it. How many of those now in practice could even comply with the requirements of the better schools? Suppose the members of the various Examining Boards were themselves required to pass an examination one-half as rigid as that which they exact of candidates for practice; how many could do this? It is easy to look out questions from some text book, the elementary principles of which the querist himself may not comprehend, but if he were required to pass the college examinations, could he succeed? We venture the statement that not one in ten of the old practitioners could comply with the modern demands; and yet some of them are eminent in their profession. The truth is, dentistry has developed greatly since they entered upon its practice. It is daily developing, but its educational standard cannot be placed too far in advance of its real status without checking instead of fostering that development.

We do not mean to imply that the proposed requirements are greater than they should be. They are far less than is demanded by the State of New York. But the schools must all be brought along together. The whole line must advance. If in action a single company pushes far ahead of the rest, and takes up a position in which it is unsupported by the rest of the army, it will itself be cut off. Hence those who are struggling for the best interests of dentistry are attempting to secure a general forward movement. While they are laboring earnestly in this direction, it is disheartening to see all their efforts made nugatory by the rantings of radicals who, occupying a side position, are unable to view the whole field, and who know nothing of the obstacles in the way. It is not pleasant to be classed as an obstructionist when one knows that he is working in the only direction that can bring ultimate success.

The members of the Faculties Association are as honest as other men. They as earnestly desire the elevation of their profession as do any members of the Examiners Association. They are no more likely to be swayed by selfish considerations than are those who are so determinedly struggling for place and power and domination over their brethern and peers. They welcomed the organization of Examining Boards, and extended to them the helping hand, until at the instigation and under the influence of designing men it was sought to direct their whole power against the schools. The latter have developed faster than has dentistry itself, and even now their standard is in advance of the status of the profession, as is proved by the fact that such numbers of illiterate men are sent up to the schools by practicing dentists, and their acceptance demanded. The colleges will heartily co-operate with the Boards, but only on equal terms. They will not attempt to dictate to the Examiners, nor will their own self respect permit them to submit to insulting exactions.

Let the Examiners Association establish a standard of admission to its own ranks before it sets up one for the colleges. As it now is, one may be a member of the National Association of Dental Examiners who knows absolutely nothing of dentistry. He may be living in violation of every ethical law, and yet his acceptance be urged as an exponent of the highest interests of dentistry. Think of the absurdity of demanding that the graduates of our best colleges shall submit themselves to the examination of a man who never saw the inside of a college! With what propriety can a man examine students under, and pronounce upon the efficiency and correctness of a curriculum of which he knows nothing? He may be an excellent practitioner, and have attained to great eminence in practical matters, without any comprehension of the basal scientific principles upon which a college course is founded. He may be expert as a manipulator, and yet quite ignorant of the theories which are taught in the schools.

Such a man is not qualified to pass upon the college course of study, and it is absurd to demand of the schools that they shall submit to his dictation. We repeat: Let the Examiners establish a standard for themselves before they try to impose one on others. Let them provide that only competent men shall fill their places, and they will command a greater portion of respect than is accorded to them now. Let them refuse to receive unqualified men, who for political reasons are appointed by dirty politicians as the reward for the basest political servitude. Let them see that only clean men, having the proper qualifications, who have received their appointments at clean hands, hold positions as Examiners. Let them relegate to back seats the disturbers of the peace of our Israel, who are endeavoring to advance themselves by appealing to the basest jealousies, and by wanton misconstruction of the actions and words of honorable men. When this is done, and when the Examiners confine themselves to the duties which naturally belong to such Boards, they will not encounter opposition at the hands of any college man in existence. Our profession needs elevation, and anyone who is truly laboring for that end will welcome the assistance of every conscientious dentist.

But the very existence of the schools, their integrity, their efficiency, the welfare of the profession and the good of the public, demand that the attempt of a few ambitious, aggressive men, who have no special qualifications for the task, to assume domination over our dearest and most vital interests and offensively to dictate to those who, to say the least, are equally wise and honest with themselves, shall meet with the most determined resistance.—*Dr. Barrett in Dental Practitioner and Advertiser.*

---

ARTICLE II.

